# Cancer Incidence and Mortality in Patients with Type 2 Diabetes Treated with Human Insulin: A Cohort Study in Shanghai

**DOI:** 10.1371/journal.pone.0053411

**Published:** 2013-01-07

**Authors:** Yunjuan Gu, Chunfang Wang, Ying Zheng, Xuhong Hou, Yifei Mo, Weihui Yu, Lei Zhang, Cheng Hu, Hairong Nan, Lei Chen, Jie Li, Yuxiang Liu, Zhezhou Huang, Ming Han, Yuqian Bao, Weijian Zhong, Weiping Jia

**Affiliations:** 1 Department of Endocrinology and Metabolism, Shanghai Jiao Tong University Affiliated Sixth People’s Hospital; Shanghai Diabetes Institute; Shanghai Clinical Centre of Diabetes, Shanghai, China; 2 Department of Medicine, Shanghai Jiao Tong University School of Medicine, Shanghai, China; 3 Shanghai Key Laboratory of Diabetes Mellitus, Shanghai, China; 4 Department of Endocrinology and Metabolism, Affiliated Hospital of Nantong University, Nantong, Jiangsu Province, China; 5 Department of Vital Statistics, Shanghai Municipal Center for Disease Control and Prevention, Shanghai, China; 6 Department of Cancer Prevention and Control, Shanghai Municipal Center for Disease Control and Prevention, Shanghai, China; 7 Department of Community Medicine, School of Public Health, the University of Hong Kong, Hong Kong, China; 8 The vice-director of the Shanghai Municipal Center for Disease Control and Prevention, Shanghai, China; CUNY, United States of America

## Abstract

**Aim:**

The aim was to investigate the association between human insulin and cancer incidence and mortality in Chinese patients with type 2 diabetes.

**Methods:**

We recruited 8,774 insulin-naïve diabetes patients from the Shanghai Diabetes Registry (SDR). The follow-up rate was 85.4%. All subjects were divided into the insulin use cohort (n = 3,639) and the non-insulin use cohort (n = 5,135). The primary outcome was the first diagnosis of any cancer. The secondary outcome was all-cause mortality. Cox proportional hazards model was used to estimate the relative risk (RR) of cancer and mortality.

**Results:**

We observed 98 cancer events in the insulin use cohort and 170 in the non-insulin use cohort. Cancer incidence rates were 78.6 and 74.3 per 10,000 patients per year in the insulin users and the non-insulin users, respectively. No significant difference in cancer risk was observed between the two cohorts (adjusted RR = 1.20, 95% CI 0.89–1.62, *P = *0.228). Regarding site-specific cancers, only the risk of liver cancer was significantly higher in the insulin users compared to that in the non-insulin users (adjusted RR = 2.84, 95% CI 1.12–7.17, *P = *0.028). The risks of overall mortality (adjusted RR = 1.89, 95% CI 1.47–2.43, *P*<0.0001) and death from cancer (adjusted RR = 2.16, 95% CI 1.39–3.35, *P = *0.001) were all significantly higher in the insulin users than in the non-insulin users.

**Conclusion:**

There was no excess risk of overall cancer in patients with type 2 diabetes who were treated with human insulin. However, a significantly higher risk of liver cancer was found in these patients. Moreover, insulin users showed higher risks of overall and cancer mortality. Considering that individuals treated with insulin were more likely to be advanced diabetic patients, caution should be used in interpreting these results.

## Introduction

Diabetes and anti-diabetic therapy were both possibly related to cancer morbidity and mortality. Several cohort studies have strongly suggested that the incidence of some types of cancer and mortality is higher in patients with diabetes, predominantly type 2 diabetes [1]. There is also evidence suggests that several hypoglycemic medications are associated with either an increased or a reduced risk of cancer [2]. Insulin is one of the most commonly prescribed medications, and the interest in its role on cancer risk in diabetic patients has increased recently. Some results showed that insulin was associated with an increased cancer risk [3], [4], [5], [6], [7], while others suggested that insulin did not play a role in cancer development [8]. Similarly, there is an inconsistency with regard to the safety of insulin therapy in Chinese diabetic population. Yang et al. analyzed the database from the Hong Kong Diabetic Registry and reported that insulin was related to lower incidence of cancer [9]. Chang et al. reported that insulin glargine did not increase the overall cancer incidence when compared with human insulin in the Taiwanese population [10], and Tseng et al. found that insulin use did not increase colon cancer mortality [11]. However, studies regarding the relationship between insulin and cancer incidence and mortality in the population from Mainland China are still lacking. During the past few decades, human insulin has been widely used in diabetic population from Mainland China. Therefore, the primary objective of the present study was to survey the overall and site-specific cancer risk in Chinese patients with type 2 diabetes who were treated with human insulin. Our secondary objective was to examine the relationship between insulin use and mortality in patients with type 2 diabetes.

## Methods

### Ethics Statement

This study was approved by the institutional review board of Shanghai Jiao Tong University Affiliated Sixth People’s Hospital in accordance with the principles of the Helsinki Declaration II. Written informed consent was obtained from each participant.

### Research Design

This was a population-based, observational cohort study performed by using the Shanghai Diabetes Registry (SDR) database, which was established in December 2001 at the Shanghai Jiao Tong University Affiliated Sixth People’s Hospital, the Shanghai Clinical Center for Diabetes. This computerized database was set up with the aim of evaluating the outcome of Chinese patients with diabetes. Outpatients and hospitalized patients, who were willing to attend this follow-up program, were asked to register in our center. After filling in the registration details, each outpatient underwent a comprehensive assessment of diabetic complications. The data for the discharged patients were collected during hospitalization. Each enrolled patient was given a unique registry number and invited to attend follow-up at the center until the time of death. During follow-up, glucose-lowering agents were prescribed by trained clinical physicians based on each patient’s glucose levels. Detailed information of each subject, including demographics, diagnoses, laboratory measurements and drug prescriptions, were recorded by trained diabetic nurses.

### Cohort Definition

Patients who were insulin naïve and without a diagnosis of cancer were eligible for analysis. Those patients who initiated insulin therapy (new users) were defined as insulin users and those who received OAD (new and previous users) but no insulin therapy were defined as non-insulin users.

### Outcomes

The primary outcome was the first recorded diagnosis of any cancer. The secondary outcome was all-cause mortality. The identification of cancer events, sites, and death was performed according to the International Classification of Diseases, 9^th^ and 10^th^ revisions (ICD-9, ICD-10). The information of all cancer events, cancer sites, and causes of death was retrieved from Shanghai Municipal Center for Disease Control and Prevention.

### Patients Screening

**Figure 1:**
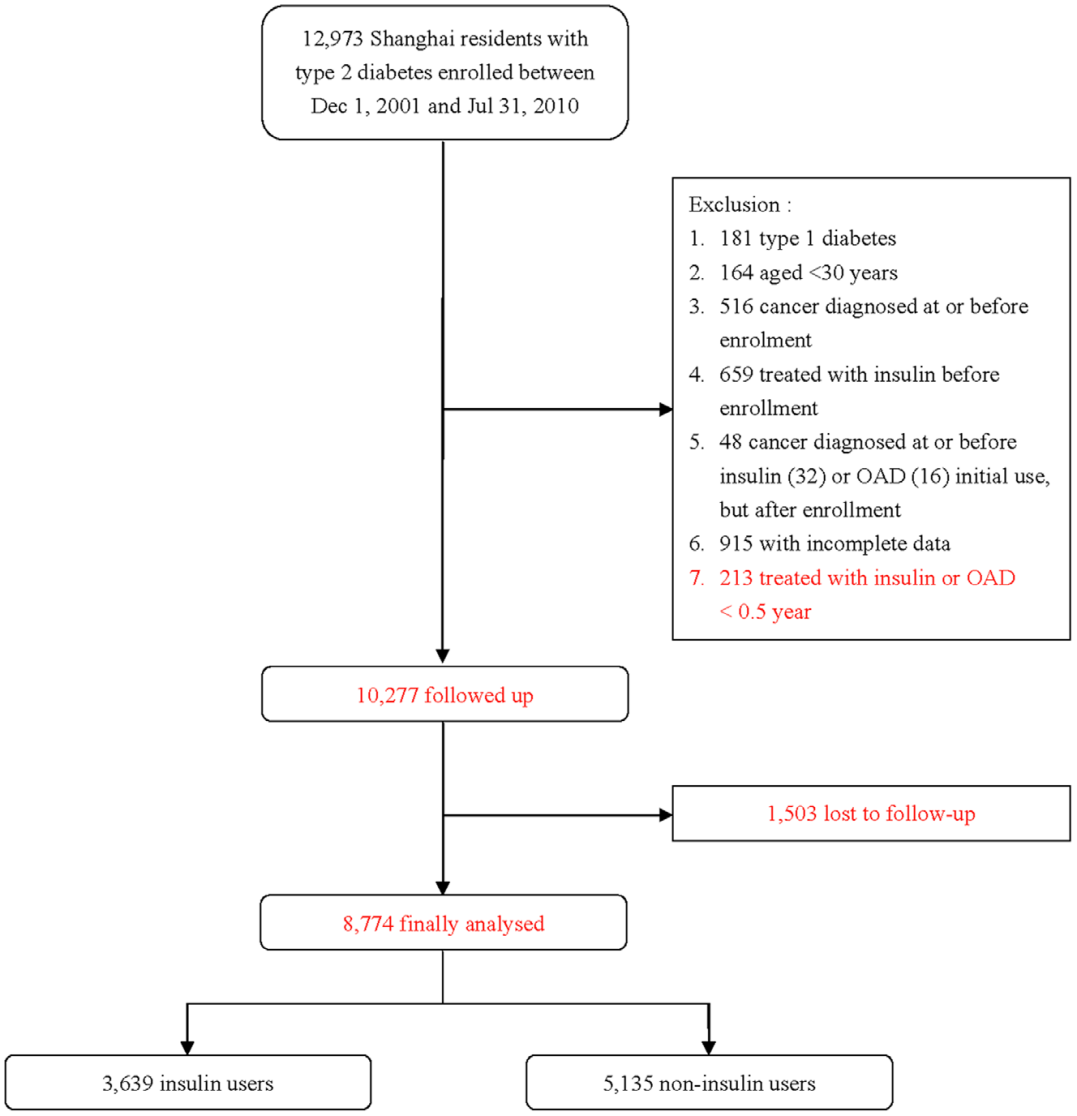
Flow chart of the type 2 diabetic patients in the study.

A total of 12,973 Shanghai residents with diabetes enrolled in our registry from December 1, 2001 to July 31, 2010 (Figure 1). There were 2696 patients excluded in the present study: including 181 patients with type 1 diabetes and 164 patients aged under 30 years; 516 patients diagnosed with cancer at or before enrollment and 659 patients treated with insulin before enrollment; 32 and 16 patients who were diagnosed with cancer before insulin or OAD initial use but after enrollment, respectively; 213 subjects treated with insulin or OAD for less than 6 months; another 915 with incomplete data. Among the remaining 10,277 patients, 1,503 were lost to follow up. The follow-up rate was 85.4%. Finally, 8,774 patients were eligible for the present analysis, including 3,639 patients in the insulin user cohort and 5,135 in the non-insulin user cohort.

### Follow-up Time

In the insulin use cohort, baseline was defined as the date of initiating insulin use. In the non-insulin use cohort, baseline was defined as the date of enrollment (previous users) or the date of OAD initially use (new users). The follow-up time for cancer outcome was calculated from baseline to the earliest date of the first cancer diagnosis, date of death if no cancer was diagnosed, or the end date of the study (July 31, 2011) if no cancer was diagnosed nor had the patient died. The follow-up time for mortality outcome was calculated from baseline to the date of any cause of death, or the end date of the study.

### Data use from the SDR

We extracted data from the computerized SDR database. The available data in the study included age, sex, body mass index (BMI), diabetes duration, waist circumference, hypertension history, smoking and drinking status, systolic blood pressure (SBP), the lipid spectrum (total cholesterol [TC], triglyceride [TG], high-density lipoprotein [HDL], and low-density lipoprotein [LDL]), glycosylated hemoglobin (HbA_1c_), fasting C-peptide, microvascular complications (nephropathy and retinopathy), and macrovascular complications (coronary artery disease, cerebrovascular disease, and peripheral artery disease).

### Statistical Analyses

All continuous variables were checked for normal distribution by the Kolmogorov–Smirnov normality test. Normally distributed variables are expressed as mean ± standard deviation, while skewed variables are expressed as the median (interquartile range, IQR). The unpaired two-tailed Student’s t-tests and Mann-Whitney U-tests were applied to examine difference between the two groups for normally and non-normally distributed parameters, respectively. The chi-square test was used to compare categorical variables between two groups. Kaplan–Meier survival curves were calculated for both cohorts and compared using the log-rank test. The Cox regression model was then conducted to adjust for potential confounders, including age, sex, diabetes duration, smoking status, HbA_1c_, macrovascular disease, and the use of oral glucose-lowering agents (including metformin, sulfonylureas and acarbose). We considered *P* values of <0.05 (2-tailed tests) to be statistically significant. Statistical analysis was performed using SPSS 17.0 (SPSS, Chicago, IL, USA).

## Results

### Baseline Characteristics

Clinical characteristics of the two inception cohorts at baseline are shown in Table 1. Compared to the non-insulin use cohort, the insulin use cohort showed older age and a lower percentage of men. The insulin users exhibited a longer median diabetic duration and a lower median level of fasting C-peptide than the non-users. Greater percentages of patients with a history of hypertension, microvascular as well as macrovascular complications were detected in the insulin use cohort. Those patients treated with insulin had lower frequencies of metformin and sulfonylureas use. Glucose control was worse in the insulin use cohort than in the non-insulin use cohort.

**Table 1:** Comparison of characteristics between insulin users and non-insulin users.

Characteristics	Insulin users n = 3639	Non-Insulin users n = 5135	*P*
Men, no. (%)	1874 (51.5)	2795(54.4)	0.007
Age at baseline, year	62.2 (18.0)	61.0 (17.0)	<0.0001
Duration of diabetes, years	9.1 (9.7)	4.0 (7.7)	<0.0001
Smoking status, no. (%)			0.695
Ex-smoker	140 (3.8)	202(4.0)	
Current smoker	697 (19.2)	1019 (19.8)	
Non-smoker	2802 (77.0)	3914 (76.2)	
Alcohol drinking status, no. (%)			0.238
Ex-drinker	316 (8.7)	494 (9.6)	
Current drinker	95 (2.6)	119 (2.3)	
Non-drinker	3228 (88.7)	4522 (88.1)	
History of hypertension, no. (%)	1804(49.6)	2426 (47.2)	0.031
SBP, mmHg	135.0 (30.0)	135.0 (26.0)	0.422
BMI, kg/m^2^	24.1 (4.3)	24.2 (4.2)	0.351
Waist circumference, cm			
Man	89.0 (12.0)	89.0 (11.0)	0.067
Woman	86.0 (13.0)	85.0 (12.0)	<0.0001
HbA_1c_, %	8.6 (3.5)	7.2 (2.2)	<0.0001
Fasting C-peptide, ng/ml	1.9 (1.5)	2.1 (1.6)	<0.0001
TC, mmol/l	4.9 (1.3)	4.9 (1.3)	0.133
TG, mmol/l	1.4 (1.0)	1.5 (1.0)	0.573
HDL, mmol/l	1.2 (0.4)	1.2 (0.4)	0.235
LDL, mmol/l	3.0 (1.3)	3.1 (1.1)	0.401
Microvascular complication, no. (%)	808 (22.2)	900 (17.5)	<0.0001
Macrovascular complication, no. (%)	917 (25.2)	752 (14.6)	<0.0001
Metformin, no. (%)	1310 (36.0)	2286 (44.5)	<0.0001
Sulfonylureas, no. (%)	1480 (40.7)	3050 (59.4)	<0.0001
Acarbose, no. (%)	2484 (68.3)	3585 (69.8)	0.120

Data are presented as median (inter-quartile range) for non-normally distributed variables and no. (%) for categorical variables, unless specified otherwise. The t-test was used for variables with normal distribution, the Wilcoxon signed ranks test for non-normal distributed variables, and the χ^2^ test for proportions. SBP: systolic blood pressure; BMI: body mass index; TC: total cholesterol; TG: triglycerides; HDL: high-density lipoprotein; LDL: low density lipoprotein.

### Incidence Rate of Cancer

The overall mean follow-up time was 48.7 months (median, 46.1 mouths; maximum, 115.3 months) for cancer incidence and 49.2 months (median, 46.5 mouths; maximum, 115.3 mouths) for mortality. The median time under observation for cancer was longer in the non-insulin use cohort than in the insulin use cohort (50.7 months *vs.* 40.4 months, *p*<0.0001). We observed 98 cancer events with an incidence rate of 76.8 per 10,000 patients per year in the insulin users and 170 cancer events with an incidence rate of 74.3 per 10,000 patients per year in the non-insulin users. As for site-specific cancers, liver cancer (11.9 per 10,000 patients per year) showed the highest incidence rate in the insulin use cohort, while lung cancer (12.0 per 10,000 patients per year) exhibited the highest incidence rate in the non-insulin use cohort.

**Figure 2:**
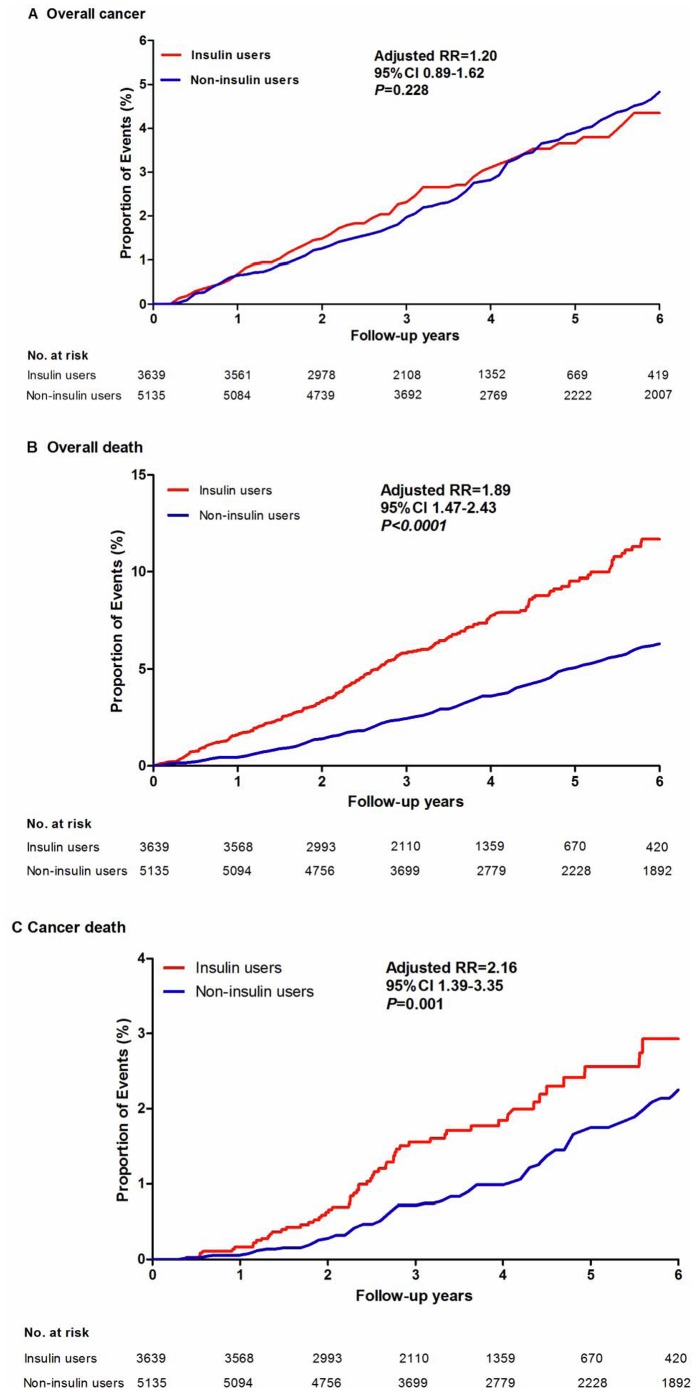
Kaplan–Meier curves of the proportion of participants with events over time.

As shown in Table 3, no significant difference was detected for overall cancer risk between the insulin users and the non-insulin users (crude RR = 1.05, 95% CI 0.82–1.35, *P = *0.695; fully adjusted: RR = 1.20, 95% CI 0.89–1.62, *P = *0.228, Figure 2A). Regarding site-specific cancers, only liver cancer risk was higher in the insulin users compared with the non-insulin users (fully adjusted RR = 2.84, 95% CI 1.12–7.17, *P = *0.028). Pancreatic cancer risk was higher in the insulin users compared with the non-insulin users in the crude model (RR = 2.55, 95% CI 1.04–6.26, *P = *0.042), but was not statistically significant after fully adjusted (RR = 2.73, 95% CI 0.93–7.99, *P = *0.067).

**Table 3:** Intermediate and final models with RRs (95% CIs) for insulin use (reference group: non-insulin use) for cancer events.

Cancer	Unadjusted	Adjusted for age and sex	Adjusted for age, sex, and smoking status	Adjusted for age, sex, smokingstatus, anddiabetesduration	Adjusted for age, sex, smoking status, diabetes duration, macrovascular, and HbA_1c_	Adjusted for age, sex, smoking status, diabetes duration, macrovascular, HbA_1c_. and concomitant oral glucose lowering agents*
Overall cancer	1.05 (0.82–1.35)	0.99 (0.77–1.28)	0.99 (0.77–1.27)	1.04 (0.80–1.35)	1.20 (0.89–1.61)	1.20 (0.89–1.62)
Breast cancer (women)	0.45 (0.15–1.37)	0.41 (0.14–1.25)	0.41 (0.14–1.25)	0.35 (0.11–1.12)	0.35 (0.10–1.18)	0.33 (0.10–1.13)
Lung cancer	0.87 (0.45–1.69)	0.80 (0.41–1.57)	0.80 (0.41–1.56)	0.97 (0.49–1.93)	0.97 (0.45–2.07)	1.00 (0.46–2.17)
Colorectal Cancer	1.49 (0.72–3.09)	1.38 (0.66–2.88)	1.37 (0.65–2.85)	1.32 (0.61–2.83)	1.28 (0.53–3.12)	1.25 (0.50–3.12)
Gastric cancer	0.70 (0.34–1.47)	0.66 (0.31–1.38)	0.65 (0.31–1.36)	0.59 (0.27–1.27)	0.57 (0.25–1.31)	0.53 (0.23–1.23)
Liver cancer	2.03 (0.95–4.35)	1.91 (0.89–4.12)	1.92 (0.89–4.13)	1.93 (0.87–4.28)	2.88 (1.16–7.16)	2.84 (1.12–7.17)
Pancreatic cancer	2.55 (1.04–6.26)	2.26 (0.92–5.58)	2.27 (0.92–5.60)	2.54 (1.00–6.45)	2.55 (0.88–7.36)	2.73 (0.93–7.99)
Prostate cancer (men)	1.37 (0.47–4.00)	1.34 (0.45–3.93)	1.36 (0.46–4.01)	1.70 (0.56–5.12)	1.97 (0.60–6.45)	2.07 (0.62–6.95)

* Oral glucose-lowering agents, including metformin, sulfonylureas, and acarbose.

### Incidence Rate of Mortality

At the end of the study, a total of 248 death events occurred in the insulin use cohort and 227 occurred in the non-insulin use cohort, with an incidence rate of 193.7 per 10,000 patients per year and 99.0 per 10,000 patients per year, respectively (Table 2). Higher overall mortality risk was observed in the patients under insulin therapy (fully adjusted RR = 1.89, 95% CI 1.47–2.43, *P*<0.0001, Table 4, Figure 2B). For the causes of mortality, risks of death from cancer (fully adjusted RR = 2.16, 95% CI 1.39–3.35, *P = *0.001, Table 2, Figure 2C) and from pneumonia and respiratory system diseases (fully adjusted RR = 2.31, 95% CI 1.27–4.18, *P = *0.006) were significantly higher in the insulin users compared with the non-insulin users. Although the incidence rate of death from cardiovascular disease was higher in the insulin users (32.8 per 10,000 patients per year) than in the non-insulin users (14.0 per 10,000 patients per year), no significant difference between the two cohorts was found for the risk of death from cardiovascular disease (fully adjusted RR = 1.83, 95% CI 0.91–3.68, *P = *0.001). The risks of death from cerebrovascular disease (fully adjusted RR = 0.83, 95% CI 0.43–1.62, *P = *0.593) and renal failure (fully adjusted RR = 2.25, 95% CI 0.63–7.95, *P = *0.210) were not significantly different between the two cohorts.

**Table 2:** Crude incidence rate of cancer and mortality among insulin users *vs*. non-insulin users.

Outcomes	Insulin users		Non-insulin users	
	No of case (%)	Incidence rate per 10,000 patients per year	No of Case (%)	Incidence rate per 10,000 patients per year
**Cancer**				
Follow-up time, months (median, IQR)	40.4 (27.8)		50.7 (42.3)	
Observed person-years	12759.0		22884.7	
Overall cancer	98 (100.0)	76.8	170 (100.0)	74.3
Breast cancer (women)	4 (4.1)	3.2	14 (8.2)	6.2
Lung cancer	13 (13.3)	10.3	27 (15.9)	12.0
Colorectal Cancer	13 (13.3)	10.3	18 (10.6)	8.0
Gastric cancer	10 (10.2)	8.0	26 (15.3)	11.6
Liver cancer	15 (15.3)	11.9	12 (7.1)	5.4
Pancreatic cancer	12 (12.2)	9.6	8 (4.7)	3.6
Prostate cancer (men)	7 (7.1)	5.6	9 (5.3)	4.1
**Mortality**				
Follow-up time, months (median, IQR)	40.6 (27.8)		51.2 (42.3)	
Observed person-years	12804.2		22930.5	
Overall mortality	248 (100.0)	193.7	227	99.0
Death from cancer	57 (23.0)	44.8	71 (31.3)	31.0
Death from cardiovascular disease	42 (16.9)	32.8	32 (14.1)	14.0
Death from cerebrovascular disease	32 (12.9)	25.0	37 (16.3)	16.1
Death from pneumonia and respiratory failure	58 (23.4)	45.3	38 (16.7)	16.6
Death from renal failure	15 (6.0)	11.7	7 (3.1)	3.1

**Table 4:** Intermediate and final models with RRs (95% CIs) for insulin use (reference group: non-insulin use) for mortality.

Mortality	Unadjusted	Adjusted forage and sex	Adjusted for age, sex, and smoking status	Adjusted for age, sex, smokingstatus, anddiabetesduration	Adjusted for age, sex, smoking status, diabetes duration, macrovascular, and HbA_1c_	Adjusted for age, sex, smoking status, diabetes duration, macrovascular, HbA_1c_, and concomitant oral glucose lowering agents*
Overall mortality	2.06 (1.72–2.48)	1.85 (1.53–2.22)	1.85 (1.53–2.21)	1.77 (1.44–2.17)	1.98 (1.55–2.54)	1.89 (1.47–2.43)
Death from cancer	1.58 (1.11–2.25)	1.46 (1.02–2.08)	1.44 (1.01–2.05)	1.60 (1.11–2.31)	2.11 (1.37–3.25)	2.16 (1.39–3.35)
Death from cardiovasculardisease	2.41 (1.51–3.85)	2.02 (1.26–3.24)	2.02 (1.26–3.24)	1.92 (1.10–3.37)	1.88 (0.95–3.71)	1.83 (0.91–3.68)
Death from cerebrovascular disease	1.61 (1.00–2.59)	1.44 (0.89–2.33)	1.43 (0.88–2.31)	1.22 (0.72–2.09)	0.89 (0.47–1.69)	0.83 (0.43–1.62)
Death from pneumonia and respiratory failure	2.98 (1.97–4.52)	2.64 (1.73–4.01)	2.65 (1.74–4.04)	2.29 (1.43–3.67)	2.43 (1.36–4.33)	2.31 (1.27–4.18)
Death from renal failure	4.05 (1.63–10.06)	3.50 (1.40–8.74)	3.54 (1.42–8.85)	2.63 (0.94–7.30)	2.52 (0.74–8.54)	2.25 (0.63–7.95)

* Oral glucose-lowering agents, including metformin, sulfonylureas, and acarbose.

## Discussion

### Insulin use and Cancer Risk

The present study showed that insulin use was not significantly associated with overall cancer risk in Chinese patients with type 2 diabetes. The result is consistent with the ORIGIN trial [12] which reported that insulin glargine had a neutral effect on cancers. Recently, a nationwide cohort study in Denmark [13] reported that use of human insulin was associated with a significantly higher rate ratio (RR = 1.40, 95% CI 1.33–1.48) compared with unexposed individuals. But the highest RR of cancer was found during the first 30 days of treatment, and declined rapidly, reaching a RR of cancer comparable to unexposed individuals after 6–12 months of therapy. Therefore, the authors suggested that the relation between higher cancer risk and human insulin use were most likely to be due to confounding and/or surveillance bias rather than causal [13].

For site-specific cancers, we found that use of insulin was only significantly associated with a higher risk of liver cancer after full adjustment. This finding was in accordance with several previous studies. Chang et al [14] found that newly diagnosed diabetic patients treated with insulin were more likely to develop solid cancers of the liver, colorectal, pancreas, lung, and stomach than those treated without insulin. Lai SW, et al [15] found that comorbidity with cirrhosis and/or hepatitis appeared to be associated with a very high increased risk of developing liver cancer among diabetic patients. They also found that liver cancer risk reduced more significantly in those diabetic patients treated with metformin or thiazolidinediones but not insulin. In terms of mechanisms of the link between insulin use and an increased risk of liver cancer, Heuson et al. suggested that liver cells are exposed to higher insulin concentrations than other tissues due to the portal circulation, a condition that is exacerbated in individuals with insulin-resistant hyperinsulinemic type 2 diabetes [16]. However, the liver is exposed to the same insulin levels as the other organs in insulin-deficient diabetic patients who were treated with exogenous insulin [17]. Thus, this assumption might only explain the relation between insulin use and higher risks of liver cancer in those patients with insulin resistance, but not insulin-deficient patients. Nonetheless, there is also evidence suggested that both mean and total volumes of hepatocellular tumors in the insulin-deficient mice were more than two-fold larger than those in the normal controls, with no significant difference in tumor number. The tumors in insulin-deficient mice showed a significantly lower frequency of apoptosis but no alteration in cell proliferation [18]. Therefore, the exact mechanisms underlying the association between a high risk of liver cancer and insulin use in type 2 diabetes are still unclear and further clinical and laboratory studies are required to clarify these mechanisms.

### Insulin use and Mortality Risk

Several studies found that insulin use was associated with risk for mortality in type 2 diabetes [3], [19]–[20]. Some of these studies compared the effects of insulin with metformin [3], while others examined the safety of insulin by comparison of human insulin-treated diabetic patients with non-diabetic patients [20]. In Bowker’s study, it was not clear whether this increased risk is related to a deleterious effect of insulin or a protective effect of metformin [3]. Recently, the results of ORIGIN trial indicated that insulin use had no effect on overall mortality in the case of glargine insulin [12]. In our study, the overall mortality, death from cancer, and death from pneumonia and respiratory conditions were significantly higher in the insulin users compared with the non-insulin users after adjustment for all potential confounders. However, these finding might also be false positive because of the following reasons: 1) the subjects in the insulin use cohort were less healthy, with older age, longer diabetes duration, poorer glucose control, and a higher percentage of microvascular and macrovascular diseases at baseline. 2) In China, insulin is seldom prescribed for newly diagnosed type 2 diabetic patients, except that they exhibit extremely high levels of plasma glucose. In general, insulin is prescribed for patients in whom OAD treatment failed, or one or more of the following concomitant diseases are present for which OAD treatment is contraindicated: OAD allergy, chronic liver or renal diseases, multiple organ failure, or some site-specific cancers. 3) The HbA_1c_ levels were higher in the insulin use cohort at both baseline and endpoint than in the non-insulin use cohort, and high glucose levels may accelerate tumor progression [21] and death [22]. 4) Other potential confounders including hypoglycemia and weight gain or edema caused by insulin should also be considered as factors that may have contributed to the higher mortality risk in the insulin use cohort. So it should be cautious to interpret the results which may be influenced by potential confounders.

### Limitations

The current study has several limitations. Firstly, the present study was not randomized and controlled because all patients in the two cohorts underwent drug therapy under real-world conditions. Thus, imbalance regarding the characteristics of the two cohorts were presented at baseline. Secondly, we were not able to analyze the hypoglycemic events because incompleteness of our data, and hypoglycemic events may be a major potential confounding factor for the analysis of association between insulin therapy and mortality risk, especially in elderly patients. Finally, we were not able to exclude other potential confounding factors that may have affected cancer or mortality events.

### Conclusion

The present study found that insulin therapy was associated with a higher relative risk of liver cancer, but not related with other site-specific cancer and overall cancer risks in patients with type 2 diabetes. Although the insulin users showed higher risks of overall and cancer mortality than the non-insulin users in this study, caution should be used in interpreting the results because individuals treated with insulin were more likely to be advanced diabetic patients. Moreover, our findings could not be directly extrapolated to other Chinese populations and should be further investigated and replicated in the future.
